# Effectiveness and cost-effectiveness of offering a chair-based yoga programme in addition to usual care in older adults with multiple long-term conditions: a pragmatic, parallel group, open label, randomised controlled trial

**DOI:** 10.3310/nihropenres.13465.1

**Published:** 2023-10-11

**Authors:** Garry Tew, Laura Wiley, Lesley Ward, Jess Hugill-Jones, Camila Maturana, Caroline Fairhurst, Kerry Bell, Laura Bissell, Alison Booth, Jenny Howsam, Valerie Mount, Tim Rapley, Sarah Ronaldson, Fiona Rose, David Torgerson, David Yates, Catherine Hewitt

**Affiliations:** 1 Institute for Health and Care Improvement, York St John University, York, North Yorkshire, YO31 7EX, UK; 2 York Trials Unit, University of York, York, North Yorkshire, YO10 5DD, UK; 3 Department of Sport, Exercise and Rehabilitation, Northumbria University, Newcastle upon Tyne, NE1 8SG, UK; 4 British Wheel of Yoga Qualifications, Sleaford, Lincolnshire, NG34 7RU, UK; 5 Public representative of the Trial Management Group, NA, UK; 6 Department of Social Work, Education and Community Wellbeing, Northumbria University, Newcastle upon Tyne, NE1 8SG, UK; 7 Department of Anaesthesia, York and Scarborough Teaching Hospitals NHS Foundation Trust, York, YO31 8HE, UK

**Keywords:** Yoga; aged; multimorbidity; health-related quality of life; randomised controlled trial

## Abstract

**Background:**

People with multiple long-term conditions are more likely to have poorer health-related quality of life (HRQOL). Yoga has the potential to improve HRQOL. Gentle Years Yoga© (GYY) is a chair-based yoga programme for older adults. We investigated the effectiveness and cost-effectiveness of the GYY programme in older adults with multiple long-term conditions.

**Methods:**

In this pragmatic, multi-site, open, randomised controlled trial, we recruited older adults aged ≥65 years with ≥2 long-term conditions from 15 primary care practices in England and Wales. Participants were randomly assigned to usual care control or a 12-week, group-based, GYY programme delivered face-to-face or online by qualified yoga teachers. The primary outcome was HRQOL (assessed with EQ-5D-5L) over 12 months. Secondary outcomes included anxiety, depression, falls, loneliness, healthcare resource use, and adverse events.

**Results:**

Between October 2019 and October 2021, 454 participants were randomly assigned to the intervention (n=240) and control (n=214) groups. Seven GYY courses were delivered face-to-face and 12 courses were delivered online. The mean number of classes attended among all intervention participants was nine (SD 4, median 10). In our intention-to-treat analysis (n=422), there was no statistically significant difference between trial groups in the primary outcome of HRQOL (adjusted difference in mean EQ-5D-5L = 0.020 [favouring intervention]; 95% CI -0.006 to 0.045, p=0.14). There were also no statistically significant differences in key secondary outcomes. No serious, related adverse events were reported. The incremental cost-effectiveness ratio was £4,546 per quality-adjusted life-year (QALY) and the intervention had a 79% probability of being cost-effective at a willingness-to-pay threshold of £20,000 per QALY.

**Conclusions:**

The offer of a 12-week chair-based yoga programme in addition to usual care did not improve HRQOL in older adults with multiple long-term conditions. However, the intervention was safe, acceptable, and probably cost-effective.

## Plain english summary

It is common for older adults to have two or more long-term health conditions. These conditions affect quality of life differently, with some people feeling well and others needing healthcare support. The Gentle Years Yoga programme is a chair-based yoga programme for older adults, including those with long-term conditions. We wanted to see how well this programme worked and if it offered good value for money for the United Kingdom’s National Health Service.

We tested whether offering a 12-week course of Gentle Years Yoga improved quality of life, and reduced anxiety, depression, falls and loneliness, in people aged 65 years and over who had two or more long-term conditions. We recruited 454 people through primary care practices across England and Wales, with 240 people selected at random to be invited to take part in the Gentle Years Yoga programme and the other 214 to continue with their usual care and not be offered Gentle Years Yoga. The average age of participants was 74 years, nearly two-thirds were female, and the number of long-term health conditions participants had ranged from two to nine (average was three). They completed four questionnaires over a 12-month period. We also interviewed some of the participants and the yoga teachers to find out how the approach worked in practice. The yoga was delivered either face-to-face or online.

We did not find any significant benefits in terms of quality of life, anxiety, depression, falls, or loneliness. At interview, some yoga participants noted no or a modest impact on their health or lifestyle, while others described Gentle Years Yoga as transformative, having substantial impacts and improvements on their physical health and emotional wellbeing. Because running the yoga classes was relatively inexpensive, and some insignificant benefits were seen, the Gentle Years Yoga programme might be good value for money.

## Introduction

Multiple long-term health conditions (MLTC; also known as multimorbidity), defined as the coexistence of two or more long-term conditions ^1^, is a growing global health challenge that is highly prevalent in older adults ^2, 3^. In 2015, 54% of people aged 65 years and over in England had MLTC; this is expected to reach 68% by 2035 ^3^. MLTC is associated with poorer outcomes such as reduced health-related quality of life (HRQOL), impaired functional status, worse physical and mental health, and premature death ^4, 5^. It also increases healthcare utilisation and associated costs ^6, 7^.

The evidence base for improving outcomes in people with MLTC is limited ^8, 9^. A Cochrane review found few randomised trials of interventions, with many remaining uncertainties about their effects on a range of outcomes ^9^. Evidence from elsewhere highlights yoga as a candidate intervention for improving health outcomes in this population ^10– 16^. Yoga is a mind-body practice that typically involves a combination of physical postures, breathing exercises, and concentration/meditation. It has become a popular means of promoting physical and mental wellbeing ^17^ and has been reported to improve HRQOL in older adults ^11^. However, robust evidence of clinical and cost-effectiveness is limited, and little research has specifically focused on older adults with MLTC.

The British Wheel of Yoga’s chair-based Gentle Years Yoga© (GYY) ( https://www.bwy.org.uk/gentleyearsyoga/) programme was developed to cater specifically for the needs of older adults, including those with conditions common to an older cohort such as osteoarthritis, hypertension, and cognitive impairment. A pilot randomised trial of the GYY programme (n=52 adults, mean age 75 years) demonstrated feasibility of evaluating this intervention in a full-scale randomised trial and the potential for a beneficial effect on health status (EQ-5D-5L utility index score) at three months after randomisation (mean difference 0.12, 95% confidence interval [CI] 0.03 to 0.21) ^18^. Consequently, we conducted this larger trial, the primary objective of which was to establish if the offer of a free 12-week GYY programme in addition to usual care is more effective compared with usual care alone in improving HRQOL over 12 months in community-dwelling adults aged 65 years and over with MLTC. We also aimed to assess the cost-effectiveness of the intervention in terms of quality adjusted life years and costs from a combined healthcare provider and personal social services perspective.

## Methods

### Ethical statement

This study received ethical approval from the UK’s National Research Ethics Committee North East – York under approval number/ (24/04/2019; 19/NE/0072). All participants provided written informed consent.

### Trial design

This was a pragmatic, parallel group, multi-site, open, randomised controlled trial. The protocol has been published ^19^ and the statistical and health economics analysis plans are available as extended data. Conduct and reporting followed CONSORT and CHEERS guidelines. The completed CONSORT and CHEERS checklists are available as extended data ^20^. The trial was prospectively registered on the ISRCTN registry (ISRCTN13567538).

### Participants

15 primary care practices were recruited from nine areas: Banbury, Bristol, Harrogate, Hull, Kent, Oxford, Wantage, and Wirral in England, and Newport in Wales. At each practice, an electronic database (SystmOne or EMIS) was used to identify individuals aged 65 years or older who had two or more long-term conditions from those included in the UK Quality and Outcomes Framework pay-for-performance programme. Potentially eligible patients were sent an invitation pack by Docmail (a third-party information handler). Individuals who were interested in participating were asked to return a consent form and screening questionnaire to York Trials Unit (YTU), University of York.

Trial coordinators assessed eligibility against the following criteria. Inclusion criteria were age 65 years or older, community-dwelling, and at least two types of long-term condition. Exclusion criteria were: inability to attend at least nine out of 12 classes in a GYY course on offer, yoga practice in the previous six months, medical contraindications to yoga participation, severe mental illness, learning disability, unable to provide informed consent, and unable to complete and return the baseline questionnaire. Some of the 12-week GYY courses were delivered face-to-face, and some were delivered online via Zoom. For online courses, exclusions also included no internet access, unable to use the internet, no suitable electronic device, insufficient space at home, and no sturdy chair for use during the classes. The health-related criteria were confirmed by participants’ general practitioners.

### Randomisation and blinding

Participants were randomised using a central, computer-based randomisation system, designed and managed by YTU. The randomisation was stratified by site and used varying block sizes and allocation ratios to ensure class lists were optimised. When enough patients (ideally 20–30) had provided baseline data and confirmed their availability for a specific GYY course, they were randomised collectively as a ‘batch’ (in a single block) by a member of the research team using the randomisation system. The participants were allocated either to intervention or control in a ratio that was variable to ensure that each GYY course was full to begin with (12–15 participants randomised to the intervention group, and the rest to control). We targeted an overall allocation ratio of 1:1. In all, participants were randomised in 19 batches (median 24 participants per batch, range 16 to 35). Since a group of participants were randomised simultaneously, the allocation sequence could not be predicted in advance. Randomisation occurred close to the course start date (maximum 3 weeks before) but allowed time for course planning. Participants and yoga teachers were informed of the allocation by the research team. Outcome measures were self-reported, except for details of participants’ medications which were provided by their primary care practices. Practices were not informed of allocations.

### Interventions

Participants randomised to the intervention group were offered a free GYY course involving 12 weekly group-based GYY classes and encouragement to practice yoga independently on most days. The courses were delivered either face-to-face in a non-medical community-based facility or online via Zoom video conferencing during periods of social distancing restrictions resulting from the COVID-19 pandemic. All teachers had the BWYQ Level 4 Teaching GYY qualification, appropriate insurance, and experience of working with older adults. They had also received trial standardisation training from the research team via a one-day interactive workshop and provision of a research training manual.

**Figure 1.**
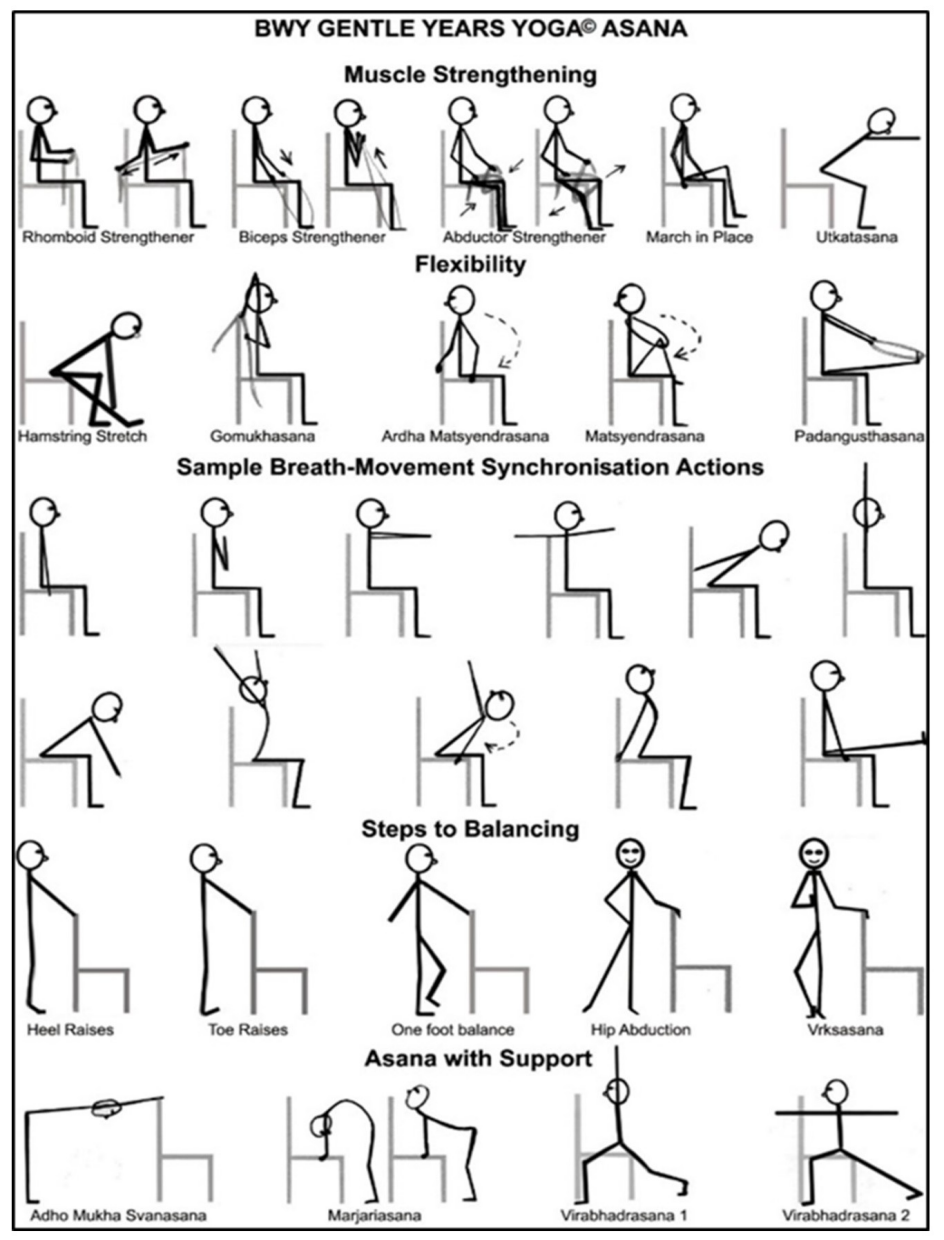
Chair-based postures that are commonly used in GYY. Reproduced from Tew *et al*. ^18^.

The aims of the chair-based GYY style of yoga are to improve muscle strength, flexibility, balance, mobility, and mental and social wellbeing. Chairs are used for seated exercise and for support when standing, although all the yoga content can be carried out while seated. Figure 1 shows examples of seated postures commonly used. The yoga practices are modified for the safety of individuals with varying medical conditions and functional abilities. Props are used to modify some of the postures and concentration activities. The physical challenge of each posture can be progressed throughout the course as participants become more able and confident. Each GYY class lasted 75 minutes and included: ‘housekeeping’ activities, five minutes; an introduction to the theme and practices of the class, basic breathing and focusing activities, five minutes; an extended warm up/mobilisation and preparatory postures, 30–35 minutes; focused postures and restorative activities, 10–15 minutes; breathing exercises, 5–10 minutes; and relaxation and concentration activities, 5–10 minutes. These activities were followed by optional after-class social time for 15–30 minutes.

Home practice sheets were distributed in four classes over the 12-week course. Each sheet included at least five yoga practices, providing an expected practice time of 10–20 minutes per session. Towards the end of the course, participants received verbal advice about continuing yoga practice and a paper or electronic handout sign-posting them to suitable yoga classes (e.g., GYY or similar) in their local community or online, which they could attend on a self-pay basis.

To assess treatment fidelity, each yoga teacher underwent an observation of one of their trial classes by one of the originators of the GYY programme (LB and JH). A fidelity check assessment form was completed for each observation and sent to the trial coordinators for review. The fidelity of content was verified by this process, and no changes resulted from the monitoring sessions.

The comparator was usual care alone. Throughout the trial, both groups continued with any usual care provided by primary, secondary, community and social services independent of the trial.

### Outcomes

The primary outcome was HRQOL measured using the EQ-5D-5L utility index score ^21^ over 12 months. Secondary outcomes were assessed at three, six, and 12 months and included HRQOL (EQ-5D-5L utility index score, EQ-5D-5L Visual Analogue Scale and PROMIS-29 v2.1) ^22^, depression severity (Patient Health Questionnaire-8) ^23^, anxiety severity (Generalized Anxiety Disorder-7) ^24^, and loneliness (three-item loneliness scale ^25^, and a direct question about how often the respondent felt lonely). The incidence of falls over 12 months was assessed via self-report. Adverse events were recorded.

Data collection and monitoring was coordinated by YTU. Outcomes were self-reported by the participant and collected using postal questionnaires at baseline and follow-up. Between April and May 2020, follow-up questionnaire data were collected by telephone due to COVID-19 restrictions.

The economic analysis outcome was the quality-adjusted life-year ^26^ over 12 months, calculated using the EQ-5D-5L. Resources were valued in 2020-21 UK prices. The methods used to estimate utility values, and measure and value resources are available as extended data.

### Sample size

The original sample size was 586 participants (293 per trial group). This number gave 90% power at 5% significance with 20% attrition to detect a clinically important difference of 0.06 in EQ-5D-5L utility index score, assuming a standard deviation of 0.20 ^18^. In October 2021, an interim calculation of the correlation between baseline and 12-month EQ-5D-5L utility index score indicated we would be able to detect this clinically important difference with close to or greater than 90% power with 454 participants, since the primary analysis adjusted for baseline score, which affords gains in power.

### Statistical analysis

The Statistical Analysis Plan is available as extended data ^20^. Analyses were conducted using Stata v17. R (RRID: SCR_001905), a freely accessible software, is also capable of the same analysis used in this study. Outcomes were analysed under the principles of intention to treat. Statistical tests were two-sided at the 5% significance level and 95% confidence intervals (CIs) and p-values were used. The flow of participants through the trial is detailed in a CONSORT diagram. All participant baseline data are summarised descriptively by trial arm both as randomised and as included in the primary analysis.

The primary outcome (HRQOL measured by EQ-5D-5L utility index score) was included in a linear mixed effects model incorporating the outcome at all post-randomisation time points and adjusting for baseline EQ-5D-5L utility index score, time point, trial arm, and trial arm by time interaction as fixed effects, and participant and site as random effects. An unstructured covariance pattern was used as this resulted in the lowest Akaike’s information criterion. The adjusted mean difference in EQ-5D-5L utility index score is presented with its 95% CI and p-value for each time point and overall. Prespecified sensitivity analyses were conducted for the primary analysis by including further adjustments for age, gender and adapted Bayliss score; and adjusting for yoga teacher as a random effect instead of trial site. Complier average causal effect (CACE) analyses were undertaken to explore the impact of non-compliance on treatment effect estimates, defining compliance as: attendance at three or more of the first six sessions and at least three other sessions; attendance at one or more yoga sessions; and number of sessions attended in its continuous form. Two-stage least squares instrumental variable regression for the EQ-5D-5L at 12 months was used, with randomised group as the instrumental variable and robust standard errors to account for clustering within site and adjusting for gender (in the first stage) since gender was thought to be associated with attendance. An exploratory subgroup analysis was conducted for mode of intervention delivery (online or face-to-face).

Secondary outcomes (EQ-5D VAS, GAD-7, PHQ-8, T-scores from each of the seven subscales of the PROMIS-29 v2.1, the physical and mental health component score and the global item score, UCLA-3, ELSA single-item direct loneliness question) were analysed as described for the primary outcome, adjusting for the baseline value of the outcome in place of baseline EQ-5D-5L utility index score. The incidence of falls over 12 months was analysed by a mixed effect negative binomial regression model, adjusting for the number of falls in the three months prior to baseline and including site as a random effect and an exposure variable for the number of months for which the participant provided falls data.

Serious and non-serious adverse events that were deemed at least possibly related to the study were summarised descriptively.

### Cost-effectiveness analysis

The Health Economics Analysis Plan is available as extended data ^20^. A within-trial economic evaluation assessed the cost-effectiveness of the GYY programme relative to usual care from the perspective of the NHS and personal social services in terms of the incremental cost per quality-adjusted life year (QALY), over a 12-month time horizon; hence discounting of costs and outcomes was not necessary. A cost-consequence analysis was also conducted to present disaggregated costs alongside all outcomes.

QALYs at 12 months were estimated using the area under the curve approach ^27^, based on responses from the EQ-5D-5L provided at baseline, three, six and 12 months. Resource use data were collected within primary care and the community, and also the hospital setting; with private treatment data collected for a sensitivity analysis. Mean resource use per participant was presented by item and group. Resource use for each item was multiplied by the corresponding unit cost, with unit costs obtained from established costing sources ^28, 29^ and costs evaluated in 2020-21 UK prices (£). Medication costs were also included based on a sample of prescription data collected from GP practices to estimate the average medication cost per participant over a 12-month period; attaching costs from the British National Formulary ^30^. The cost of the intervention comprised the cost of training yoga teachers and the cost of running the yoga classes, including equipment costs.

Multiple imputation by chained equations was used to deal with missing data, with predictive mean matching. Seemingly unrelated regression was used to estimate mean differences in costs and QALYs, with 95% CIs estimated using bias corrected and accelerated bootstrap methods. The analysis used the £20,000 willingness-to-pay threshold recommended by the National Institute for Health and Care Excellence (NICE) in the UK ^26^, for the incremental cost per QALY and for the incremental net monetary benefit estimate. Cost-effectiveness acceptability curves ^31^ exploring the probability of the intervention being cost-effective at different willingness-to-pay thresholds and sensitivity analyses were undertaken to investigate uncertainty around the cost-effectiveness findings, including a complete case analysis.

### Process evaluation

A qualitative process evaluation was undertaken to identify, describe, and explain the determinants of GYY delivery, trial processes and participant experience. Data were collected throughout the trial, from January 2020 to April 2022, via qualitative interviews with a subset of purposively sampled trial participants, trial decliners, and yoga teachers, as well as from observations of standardisation training sessions and yoga classes. A further subset of trial participants and yoga teachers took part in a second interview to explore any longer-term impact of their trial participation. The interviews and observations were conducted by an experienced qualitative researcher (LW), who, as necessitated by the purposive sampling strategy, was unblinded to allocation. Interview topic guides were developed and used however the interviews were flexible to accommodate additional unanticipated areas, the developing analysis, and in the case of follow-up interviews, what was known from the prior interview. All participants provided written informed consent for the interviews and/or observations, additional to main trial consent. The majority of interviews and observations were conducted remotely by telephone or video conference. Interviews were, with consent, audio-recorded, transcribed verbatim and edited to ensure anonymity of respondent and field notes edited to ensure anonymity. Data analysis was iterative throughout the trial and conducted according to the standard procedures of rigorous qualitative analysis ^32^. The process evaluation methods will be reported in full elsewhere.

### Patient and public involvement

In May 2018, the planned research was discussed with seven older adults with MLTC who had participated in the North Yorkshire pilot trial ^18^. The group agreed that the study was valuable and gave views on the design that shaped the protocol. Subsequently one member of this group served on the trial management group, and two other members served on the independent trial steering committee for this study.

## Results

**Figure 2.**
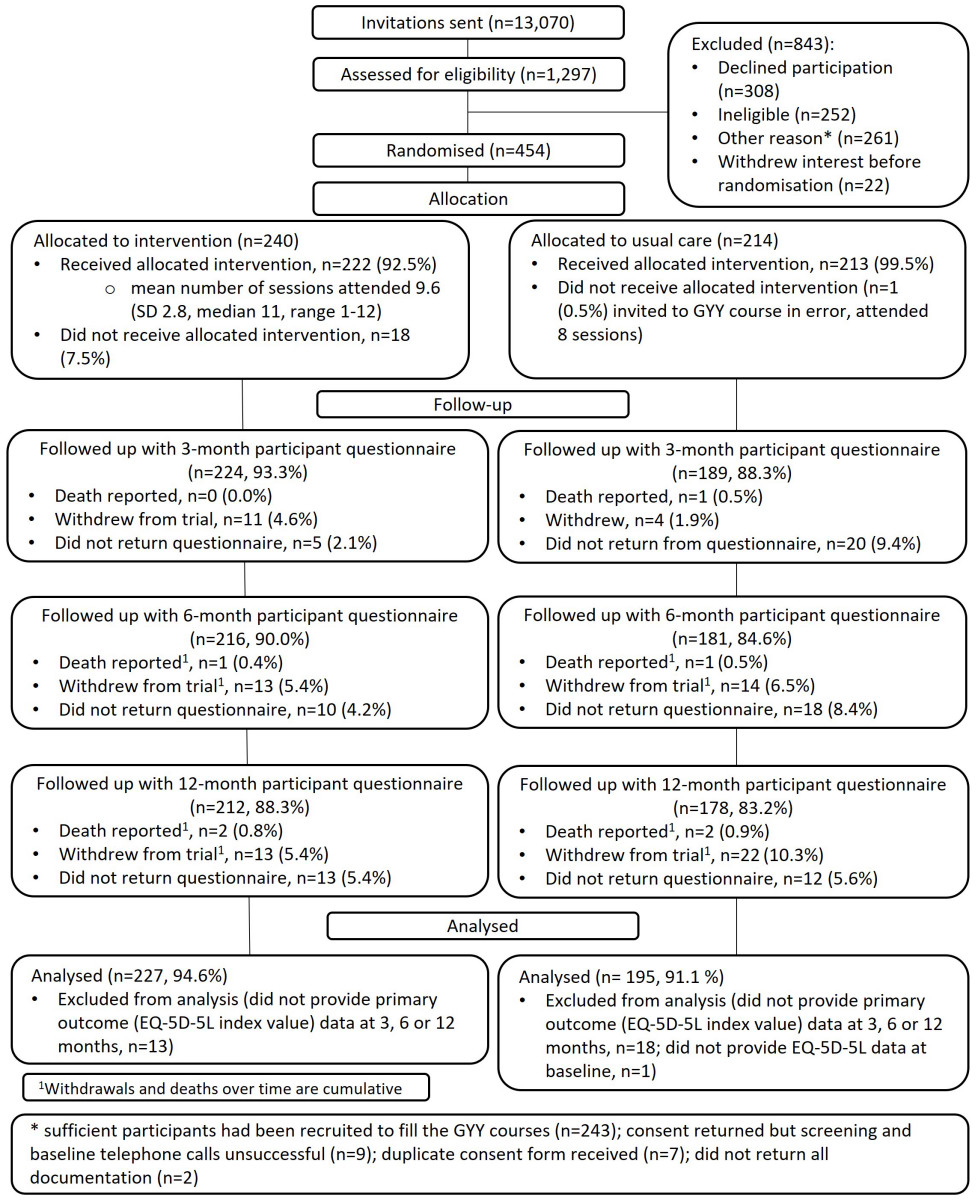
CONSORT trial diagram.

Between July 2019 and August 2021, 13,070 people from 15 primary care practices were invited to participate in the study. Out of 1,297 (9.9%) responses, 454 (3.5% of all invited) individuals were eligible, consented to take part, and were randomised (between October 2019 and October 2021) to either intervention (n=240) or control (n=214) ( Figure 2). The last participant follow-up was in October 2022.

The participants in the two groups had similar baseline characteristics ( Table 1), except that there was a slightly higher proportion of females in the intervention group (64.2% versus 56.5%). The mean age was 73.5 years (standard deviation 6.2), 60.6% were female, and the median number of long-term health conditions was three. Two thirds of participants had a cardiovascular condition (n=307 participants, 67.6%), over half had some form of arthritis (n=242, 53.3%), over a third had a severe problem with hearing or vision (n=168, 37.0%), and approximately a quarter had anxiety or depression (n=110, 24.2%) or asthma or chronic obstructive pulmonary disease (n=109, 24.0%) ( Table 2). At baseline, three quarters of participants (n=339, 74.7%) said they would prefer to be allocated to the intervention group rather than usual care alone. Most of the remaining participants had no preference (n=103, 22.7%), and only a small number preferred usual care (n=12, 2.6%).

**Table 1.** Baseline characteristics of participants as randomised and as included in the primary analysis. Data are mean (SD) unless otherwise stated.

Characteristics	As randomised			As analysed		
	Intervention (n=240)	Control (n=214)	Overall (n=454)	Intervention (n=227)	Usual care (n=195)	Overall (n=422)
Age (years)	73.4 (6.0)	73.5 (6.4)	73.5 (6.2)	73.2 (5.9)	73.4 (6.2)	73.3 (6.0)
Female sex, n (%)	154 (64.2)	121 (56.5)	275 (60.6)	143 (63.0)	105 (53.8)	248 (58.8)
Ethnic group, n (%)						
White	237 (98.7)	209 (97.7)	446 (98.2)	217 (95.6)	186 (95.4)	403 (95.5)
Other or missing	3 (1.3)	5 (2.3)	8 (1.8)	1 (0.4)	2 (1.0)	3 (0.7)
Employment status, n (%)						
Retired	219 (91.2)	196 (91.6)	415 (91.4)	208 (91.6)	178 (91.3)	386 (91.5)
Other or missing	21 (8.8)	18 (8.4)	39 (8.6)	19 (8.4)	17 (8.7)	36 (8.5)
IMD decile	7.6 (2.6)	7.5 (2.7)	7.5 (2.7)	7.7 (2.6)	7.4 (2.7)	7.5 (2.7)
Smoking status, n (%)						
Yes	5 (2.1)	5 (2.3)	10 (2.2)	5 (2.2)	5 (2.6)	10 (2.4)
No, never smoked	115 (47.9)	109 (50.9)	224 (49.3)	109 (48.0)	103 (52.8)	212 (50.2)
No, used to smoke	120 (50.0)	100 (46.7)	220 (48.5)	113 (49.8)	87 (44.6)	200 (47.4)
Number of conditions, median (range)	3 (2, 9)	3 (2, 7)	3 (2, 9)	3.0 (2.0, 9.0)	3.0 (2.0, 7.0)	3.0 (2.0, 9.0)
Bayliss illness burden score	9.6 (6.5)	9.7 (7.6)	9.7 (7.1)	9.4 (6.4)	9.7 (7.7)	9.6 (7.0)
**Outcome measures**						
EQ-5D-5L utility index score ^a^	0.742 (0.176)	0.736 (0.162)	0.739 (0.169)	0.742 (0.175)	0.736 (0.163)	0.739 (0.169)
EQ-5D VAS ^a^	75.0 (18.2)	73.4 (17.6)	74.3 (17.9)	75.4 (18.2)	73.9 (17.2)	74.7 (17.7)
PHQ-8 ^b^	3.7 (3.9)	3.8 (4.3)	3.8 (4.1)	3.6 (3.8)	3.7 (4.2)	3.7 (4.0)
GAD-7 ^b^	2.5 (3.4)	2.7 (3.6)	2.6 (3.5)	2.4 (3.3)	2.6 (3.6)	2.5 (3.4)
UCLA-3 loneliness ^b^	4.2 (1.7)	4.4 (1.9)	4.3 (1.8)	4.2 (1.7)	4.3 (1.8)	4.2 (1.7)
ELSA single-item direct loneliness question ^b^, n (%)	2.2 (1.3)	2.3 (1.3)	2.2 (1.3)	2.1 (1.3)	2.2 (1.3)	2.2 (1.3)
PROMIS-29 Physical Function ^a^	46.7 (8.5)	46.3 (8.5)	46.5 (8.5)	47.0 (8.4)	46.4 (8.4)	46.7 (8.4)
PROMIS-29 Anxiety ^b^	46.9 (8.0)	48.1 (8.5)	47.5 (8.2)	46.9 (8.0)	48.0 (8.6)	47.4 (8.3)
PROMIS-29 Depression ^b^	46.4 (7.6)	46.8 (8.1)	46.6 (7.8)	46.4 (7.5)	46.5 (8.0)	46.4 (7.7)
PROMIS-29 Fatigue ^b^	47.4 (9.7)	48.7 (9.8)	48.0 (9.8)	47.3 (9.7)	48.4 (9.8)	47.8 (9.7)
PROMIS-29 Sleep Disturbance ^b^	49.1 (9.5)	49.8 (9.6)	49.5 (9.6)	49.1 (9.6)	49.5 (9.5)	49.3 (9.5)
PROMIS-29 Social Participation ^a^	54.7 (9.3)	54.1 (9.9)	54.4 (9.6)	54.8 (9.2)	54.3 (10.0)	54.6 (9.6)
PROMIS-29 Pain Interference ^b^	53.3 (8.7)	53.6 (8.9)	53.5 (8.8)	53.2 (8.7)	53.6 (8.9)	53.4 (8.8)
PROMIS-29 Pain Intensity ^b^	3.1 (2.5)	3.2 (2.4)	3.1 (2.4)	3.1 (2.5)	3.1 (2.4)	3.1 (2.4)
PROMIS-29 physical health summary score ^a^	47.6 (8.8)	47.1 (8.8)	47.4 (8.8)	47.9 (8.6)	47.2 (8.7)	47.6 (8.7)
PROMIS-29 mental health summary score ^a^	52.9 (8.0)	52.0 (8.5)	52.5 (8.2)	53.0 (7.9)	52.2 (8.4)	52.6 (8.1)
Fallen in past 3 months, n (%)	61 (25.4)	49 (22.9)	110 (24.2)	58 (25.6)	46 (23.6)	104 (24.6)

IMD = Index of Multiple Deprivation; VAS = Visual Analogue Scale ^a^higher score indicates better outcome; ^b^lower score indicates better outcome

**Table 2.** Self-reported long-term conditions at baseline by randomised group. Data are number (%) of participants.

Long-term condition	Intervention (n=240)	Control (n=214)	Overall (n=454)
Cardiovascular disease	162 (67.5)	145 (67.8)	307 (67.6)
*Hypertension*	*132 (55.0)*	*119 (55.6)*	*251 (55.3)*
*Coronary heart disease including angina, history* *of heart attack, bypass surgery or angioplasty*	*32 (13.3)*	*36 (16.8)*	*68 (15.0)*
*Heart failure*	*15 (6.3)*	*8 (3.7)*	*23 (5.1)*
*Peripheral artery disease*	*22 (9.2)*	*19 (8.9)*	*41 (9.0)*
Arthritis	135 (56.3)	107 (50.0)	242 (53.3)
*Osteoarthritis of the shoulder, hip or knee*	*123 (51.2)*	*99 (46.3)*	*222 (48.9)*
*Rheumatoid arthritis of the shoulder, hip or knee*	*19 (7.9)*	*16 (7.5)*	*35 (7.7)*
Sensory conditions	90 (37.5)	78 (36.4)	168 (37.0)
*Deafness or severe problem with hearing*	*74 (30.8)*	*63 (29.4)*	*137 (30.2)*
*Blindness or severe problem with vision*	*30 (12.5)*	*23 (10.7)*	*53 (11.7)*
Depression or anxiety	63 (26.3)	47 (22.0)	110 (24.2)
*Anxiety*	*45 (18.8)*	*36 (16.8)*	*81 (17.8)*
*Depression*	*48 (20.0)*	*31 (14.5)*	*79 (17.4)*
Asthma or COPD	62 (25.8)	47 (22.0)	109 (24.0)
*Asthma*	*47 (19.6)*	*36 (16.8)*	*83 (18.3)*
*COPD*	*21 (8.8)*	*15 (7.0)*	*36 (7.9)*
Bowel problems	55 (22.9)	36 (16.8)	91 (20.0)
Osteoporosis or osteopenia	38 (15.8)	41 (19.2)	79 (17.4)
Diabetes	36 (15.0)	37 (17.3)	73 (16.1)
Atrial fibrillation	37 (15.4)	35 (16.4)	72 (15.9)
Cancer (last 5 years)	35 (14.6)	35 (16.4)	70 (15.4)
Chronic kidney disease	14 (5.8)	15 (7.0)	29 (6.4)
Stroke (last 5 years)	6 (2.5)	10 (4.7)	16 (3.5)
Fibromyalgia	8 (3.3)	7 (3.3)	15 (3.3)
Epilepsy	1 (0.4)	5 (2.3)	6 (1.3)
Multiple Sclerosis	3 (1.3)	2 (0.9)	5 (1.1)
Parkinson’s disease	1 (0.4)	4 (1.9)	5 (1.1)
Dementia	2 (0.8)	1 (0.5)	3 (0.7)

COPD=Chronic obstructive pulmonary disease Long-term conditions are presented as grouped according to the trial eligibility criteria, and then broken down (text in italics) by individual condition

Nineteen 12-week GYY courses were delivered in total across four ‘waves’: wave one, four face-to-face courses running from September 2019 to January 2020; wave two, four online courses running from September 2020 to January 2021; wave three, three online classes running May 2021 to September 2021; and wave four, three face-to-face and five online courses running September 2021 to January 2022. The 19 courses were delivered by 12 yoga teachers; one teacher delivered three courses, five teachers delivered two courses each, and six teachers delivered one course each. 12 participants were randomised to every online course, and either 12 or 15 (median 15) participants to every face-to-face course. The first class in a 12-week course occurred a median of 19 days after randomisation and subsequent classes were scheduled a median of seven days apart. Among the intervention group, 222 (92.5%) participants attended at least one yoga class, while 53 (22.1%) attended all 12. The mean number of classes attended among all randomised yoga participants was 8.8 (SD 3.7, median 10), and 9.6 (SD 2.8, median 11) among those who attended at least one yoga class. Eighty percent (n=192) of participants attended at least six classes, including three or more of the first six. At three months, 185 (82.6%) of intervention participants reported having practiced yoga at home in the past three months for a median of four weekly sessions and a median of 15 minutes per session. At 12 months, 55 (25.9%) intervention participants reported having attended yoga classes (GYY or other) on a self-funded basis in the previous six months and 102 (48.1%) reported having practiced yoga at home in the past six months for a median of three weekly sessions and a median of 15 minutes per session.

One participant in the control group was invited to attend trial yoga classes in error; they attended eight classes. At three months, four (2.1%) other control participants reported having attended non-trial group-based yoga classes in the previous three months and six (3.2%) reported having practiced yoga at home in the past three months for a median of two weekly sessions and a median of 15 minutes per session. At 12 months, nine (5%) control participants reported having attended yoga classes (GYY or other) on a self-funded basis in the previous six months and 17 (9.6%) reported having practiced yoga at home in the past six months for a median of two weekly sessions and a median of 10 minutes per session.

### Primary outcome measure

**Figure 3.**
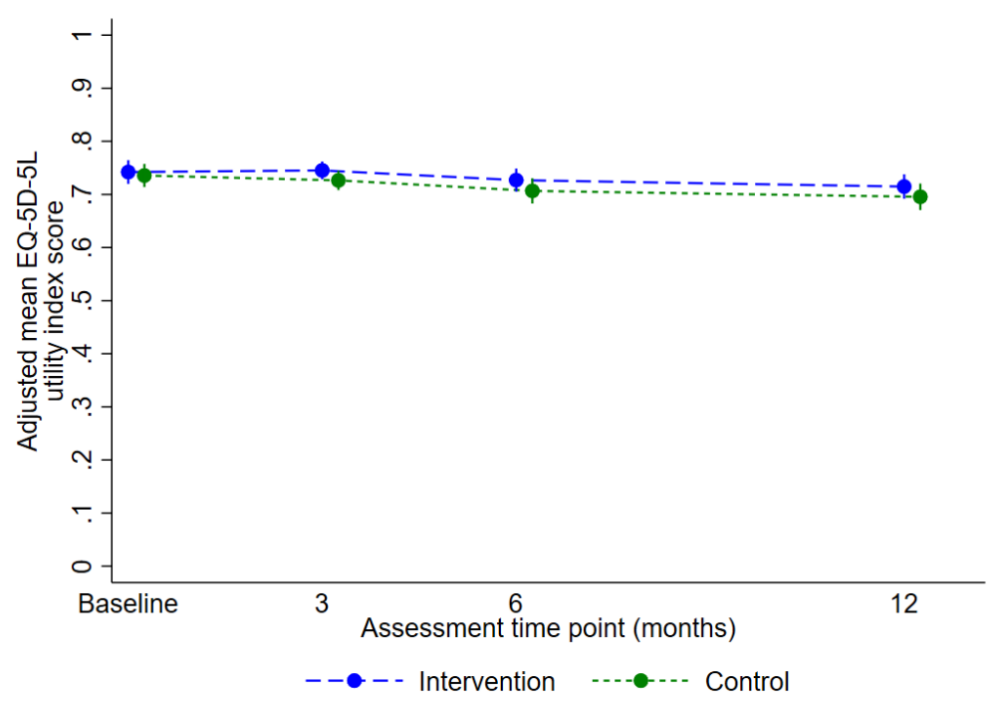
Adjusted mean (95% confidence interval) EQ-5D-5L utility index scores for primary analysis over time by randomised group.

The primary analysis included 422 participants with valid EQ-5D-5L data at baseline and at least one post-randomisation time point (intervention n=227 of 240, 94.6%; usual care n=195 of 214, 91.1%). There was no statistically or clinically significant difference in the EQ-5D-5L utility index score over 12 months: the predicted mean score for the intervention group was 0.729 (95% CI 0.712 to 0.747) and for control was 0.710 (95% CI 0.691 to 0.729); the adjusted mean difference was 0.02 favouring the intervention (95% CI -0.01 to 0.05, p=0.14) ( Figure 3, Table 3). The results were robust to sensitivity analyses ( Table 3). The CACE analyses considering compliance as attending (i) ≥1 yoga class, and (ii) ≥6 classes including three of the first six, produced slightly greater, but not clinically relevant, treatment estimates (0.025, 95% CI -0.002 to 0.052, p=0.07; and 0.029, 95% CI -0.002 to 0.059, p=0.06, respectively). The CACE estimate associated with number of sessions attended was 0.003 (95% CI -0.000 to 0.005, p=0.07). There was no evidence of an interaction between trial arm and intended mode of delivery (interaction effect 0.007, 95% CI -0.042 to 0.057, p=0.77).

**Table 3.** Difference in adjusted mean EQ-5D-5L utility index score over time by randomised group from primary and sensitivity analysis models.

Time point, months	Intervention Mean (95% CI)	Control Mean (95% CI)	Difference (95% CI)	p-value
**Primary ITT analysis**				
3	0.745 (0.728 to 0.762)	0.726 (0.708 to 0.745)	0.019 (-0.006 to 0.044)	0.14
6	0.727 (0.705 to 0.749)	0.707 (0.683 to 0.730)	0.020 (-0.012 to 0.053)	0.22
12	0.715 (0.692 to 0.738)	0.696 (0.671 to 0.720)	0.019 (-0.015 to 0.053)	0.26
Overall	0.729 (0.712 to 0.747)	0.710 (0.691 to 0.729)	0.020 (-0.006 to 0.045)	0.14
**Sensitivity analysis 1**				
3	0.745 (0.728 to 0.762)	0.727 (0.709 to 0.745)	0.018 (-0.007 to 0.042)	0.16
6	0.727 (0.705 to 0.748)	0.708 (0.684 to 0.731)	0.019 (-0.013 to 0.051)	0.24
12	0.715 (0.692 to 0.737)	0.696 (0.672 to 0.721)	0.018 (-0.015 to 0.052)	0.28
Overall	0.729 (0.712 to 0.746)	0.711 (0.692 to 0.729)	0.018 (-0.007 to 0.044)	0.16
**Sensitivity analysis 2**				
3	0.745 (0.728 to 0.762)	0.726 (0.708 to 0.745)	0.019 (-0.006 to 0.044)	0.14
6	0.727 (0.705 to 0.749)	0.707 (0.683 to 0.730)	0.020 (-0.012 to 0.053)	0.22
12	0.715 (0.692 to 0.738)	0.696 (0.671 to 0.720)	0.019 (-0.015 to 0.053)	0.26
Overall	0.729 (0.712 to 0.747)	0.710 (0.691 to 0.729)	0.020 (-0.006 to 0.045)	0.14

CI = Confidence interval Primary ITT analysis is a linear mixed effects model adjusted for baseline EQ-5D-5L utility index score, time point, trial arm, and trial arm by time interaction as fixed effects, and participant and site as random effects. Sensitivity analysis 1 is the primary analysis with further adjustment age, gender and adapted Bayliss score as fixed effects. Sensitivity analysis 2 is the primary analysis with the intended yoga teacher included as a random effect instead of site.

### Secondary outcomes

Results for measures of HRQOL, anxiety, depression and loneliness are shown in Table 4. No statistically significant differences were observed in these outcomes, except in the T-score for the pain interference subscale of the PROMIS-29 at 3 months (-1.44, 95% CI -2.63 to -0.26, p=0.02) and over the 12 months (-1.14, 95% CI -2.24 to -0.04, p=0.04), and in the global (pain intensity) PROMIS-29 item at 12 months (-0.45, 95% CI -0.83 to -0.08, p=0.02) and over the 12 months (-0.32, 95% CI -0.61 to -0.04, p=0.03), all favouring the intervention.

**Table 4.** Difference in adjusted means over time by randomised group for secondary outcomes.

Time point, months	Intervention Mean (95% CI)	Control Mean (95% CI)	Difference (95% CI)	p-value
**EQ-5D-5L VAS**				
3	75.6 (73.8, 77.4)	74.5 (72.6, 76.5)	1.08 (-1.55, 3.71)	0.42
6	73.8 (71.7, 75.9)	72.1 (69.8, 74.4)	1.74 (-1.39, 4.86)	0.28
12	73.1 (70.8, 75.3)	70.9 (68.4, 73.4)	2.18 (-1.19, 5.55)	0.20
Overall	74.2 (72.5, 75.8)	72.5 (70.7, 74.3)	1.67 (-0.78, 4.12)	0.18
**GAD-7**				
3	2.8 (2.4, 3.2)	3.0 (2.6, 3.4)	-0.17 (-0.72, 0.37)	0.53
6	2.9 (2.5, 3.3)	3.0 (2.5, 3.4)	-0.10 (-0.70, 0.50)	0.74
12	3.0 (2.5, 3.4)	2.9 (2.5, 3.4)	0.01 (-0.61, 0.63)	0.98
Overall	2.9 (2.5, 3.2)	3.0 (2.6, 3.3)	-0.09 (-0.57, 0.40)	0.72
**PHQ-8**				
3	3.9 (3.5, 4.2)	4.4 (4.0, 4.8)	-0.53 (-1.12, 0.05)	0.07
6	4.1 (3.6, 4.5)	4.4 (3.9, 4.9)	-0.30 (-0.97, 0.36)	0.37
12	4.3 (3.8, 4.7)	4.5 (4.0, 5.0)	-0.25 (-0.93, 0.43)	0.48
Overall	4.1 (3.7, 4.4)	4.4 (4.0, 4.8)	-0.36 (-0.90, 0.18)	0.19
**UCLA-3 loneliness**				
3	4.3 (4.2, 4.5)	4.3 (4.1, 4.4)	0.07 (-0.15, 0.29)	0.54
6	4.4 (4.3, 4.6)	4.4 (4.2, 4.6)	0.03 (-0.21, 0.26)	0.83
12	4.4 (4.3, 4.6)	4.4 (4.3, 4.6)	-0.00 (-0.24, 0.23)	0.97
Overall	4.4 (4.3, 4.5)	4.4 (4.2, 4.5)	0.03 (-0.16, 0.22)	0.75
**ELSA loneliness**				
3	2.3 (2.2, 2.4)	2.3 (2.2, 2.5)	-0.01 (-0.17, 0.16)	0.94
6	2.4 (2.3, 2.5)	2.3 (2.2, 2.4)	0.07 (-0.10, 0.25)	0.41
12	2.3 (2.2, 2.4)	2.4 (2.3, 2.5)	-0.10 (-0.27, 0.08)	0.28
Overall	2.3 (2.2, 2.4)	2.3 (2.2, 2.5)	-0.01 (-0.15, 0.13)	0.88
**PROMIS-29 Physical Function**				
3	46.9 (46.2, 47.6)	46.4 (45.6, 47.1)	0.50 (-0.55, 1.55)	0.35
6	46.9 (46.1, 47.7)	46.0 (45.1, 46.8)	0.90 (-0.27, 2.07)	0.13
12	46.1 (45.2, 46.9)	45.3 (44.3, 46.2)	0.80 (-0.45, 2.04)	0.21
Overall	46.6 (46.0, 47.3)	45.9 (45.2, 46.6)	0.73 (-0.22, 1.69)	0.13
**PROMIS-29 Anxiety**				
3	48.1 (47.2, 49.0)	47.3 (46.3, 48.3)	0.80 (-0.53, 2.13)	0.24
6	48.0 (47.0, 48.9)	48.3 (47.3, 49.3)	-0.35 (-1.72, 1.03)	0.62
12	48.0 (47.0, 49.1)	47.4 (46.3, 48.5)	0.67 (-0.85, 2.19)	0.39
Overall	48.0 (47.2, 48.9)	47.7 (46.8, 48.5)	0.37 (-0.82, 1.56)	0.54
**PROMIS-29 Depression**				
3	47.3 (46.4, 48.2)	47.4 (46.5, 48.4)	-0.14 (-1.42, 1.15)	0.83
6	47.9 (47.0, 48.7)	48.2 (47.3, 49.1)	-0.33 (-1.56, 0.91)	0.60
12	48.1 (47.2, 49.0)	47.4 (46.4, 48.4)	0.70 (-0.68, 2.08)	0.32
Overall	47.7 (47.0, 48.5)	47.7 (46.9, 48.5)	0.08 (-1.02, 1.17)	0.89
**PROMIS-29 Fatigue**				
3	48.1 (47.1, 49.1)	48.4 (47.3, 49.5)	-0.28 (-1.75, 1.19)	0.71
6	47.8 (46.8, 48.9)	48.8 (47.7, 49.9)	-0.97 (-2.51, 0.57)	0.22
12	49.1 (48.0, 50.1)	48.6 (47.5, 49.8)	0.43 (-1.15, 2.02)	0.59
Overall	48.3 (47.5, 49.2)	48.6 (47.7, 49.5)	-0.27 (-1.52, 0.97)	0.67
**PROMIS-29 Sleep Disturbance**				
3	50.1 (49.2, 51.0)	50.2 (49.2, 51.2)	-0.16 (-1.43, 1.11)	0.80
6	49.8 (48.9, 50.7)	50.2 (49.2, 51.2)	-0.42 (-1.68, 0.85)	0.52
12	50.0 (49.1, 50.9)	49.9 (48.9, 50.9)	0.08 (-1.22, 1.37)	0.91
Overall	50.0 (49.2, 50.7)	50.1 (49.3, 51.0)	-0.17 (-1.19, 0.85)	0.75
**PROMIS-29 Social Participation**				
3	53.0 (51.9, 54.0)	51.6 (50.5, 52.7)	1.39 (-0.14, 2.92)	0.08
6	52.3 (51.1, 53.5)	52.1 (50.8, 53.4)	0.21 (-1.60, 2.01)	0.82
12	52.4 (51.3, 53.5)	51.1 (49.9, 52.3)	1.28 (-0.37, 2.92)	0.13
Overall	52.6 (51.6, 53.5)	51.6 (50.6, 52.6)	0.96 (-0.40, 2.32)	0.17
**PROMIS-29 Pain Interference**				
3	53.0 (52.2, 53.8)	54.4 (53.5, 55.3)	-1.44 (-2.63, -0.26)	0.02
6	52.9 (52.0, 53.9)	54.0 (52.9, 55.0)	-1.03 (-2.40, 0.34)	0.14
12	53.4 (52.4, 54.4)	54.3 (53.2, 55.5)	-0.94 (-2.47, 0.59)	0.23
Overall	53.1 (52.3, 53.8)	54.2 (53.4, 55.1)	-1.14 (-2.24, -0.04)	0.04
**PROMIS-29 Pain Intensity**				
3	3.0 (2.8, 3.2)	3.3 (3.0, 3.5)	-0.26 (-0.58, 0.06)	0.11
6	3.1 (2.9, 3.4)	3.4 (3.1, 3.7)	-0.26 (-0.62, 0.09)	0.15
12	3.2 (2.9, 3.5)	3.7 (3.4, 4.0)	-0.45 (-0.83, -0.08)	0.02
Overall	3.1 (2.9, 3.3)	3.4 (3.2, 3.7)	-0.32 (-0.61, -0.04)	0.03
**PROMIS-29 Physical health summary score**				
3	47.6 (46.9, 48.3)	46.8 (46.0, 47.6)	0.76 (-0.30, 1.82)	0.16
6	47.5 (46.7, 48.3)	46.6 (45.7, 47.5)	0.89 (-0.31, 2.09)	0.14
12	46.8 (45.9, 47.7)	45.8 (44.9, 46.8)	0.97 (-0.32, 2.25)	0.14
Overall	47.3 (46.6, 48.0)	46.4 (45.7, 47.2)	0.87 (-0.11, 1.86)	0.08
**PROMIS-29 Mental health summary score**				
3	51.9 (51.2, 52.6)	51.4 (50.6, 52.2)	0.48 (-0.61, 1.56)	0.39
6	51.7 (51.0, 52.5)	51.2 (50.4, 52.1)	0.50 (-0.65, 1.66)	0.39
12	51.3 (50.5, 52.2)	51.2 (50.3, 52.1)	0.11 (-1.12, 1.35)	0.86
Overall	51.7 (51.0, 52.3)	51.3 (50.6, 52.0)	0.36 (-0.63, 1.36)	0.47

In total, 60 out of 227 (26.4%) intervention group participants and 52 out of 194 (26.8%) control group participants reported at least one fall in the follow-up questionnaires. The intervention group had a mean of 0.91 falls per person (SD 2.1, median 0, range 0 to 21) over a mean of 10.8 months (SD 3.2, median 12), whereas the control group had a mean of 0.71 falls per person (SD 1.9, median 0, range 0 to 15) over a mean of 10.2 months (SD 3.9, median 12). There was no statistically significant difference in the rate of falls between the two groups (incidence rate ratio 1.38, 95% CI 0.95 to 2.01, p=0.09).

During the trial, seven (1.5%) of 454 participants died (two (0.8%) of 240 participants in the intervention group and five (2.3%) of 214 in the control group). None of the deaths were deemed to be related to the intervention and no other serious, related adverse events were reported. There were seven non-serious adverse events for seven participants (one each) that were deemed to be at least possibly related to the intervention. These events were all new or increased musculoskeletal pain in either the back (n=3), shoulder (n=1), knee (n=1), knee and shoulder (n=1), or thigh (n=1). No event required medical attention beyond taking pain killers. Three of the seven participants withdrew from the intervention due to the pain.

### Cost-effectiveness analysis

Complete EQ-5D-5L, resource use and cost data were available for 192 (42%) participants overall, though the EQ-5D-5L had high completion rates (>83%) at all time points. Differences in resource use between the groups were small in general (extended data ^20^), though on average, higher levels of community-based care provided via GP clinics tended to be reported for the intervention group, with the exception of nursing-based care. The community service most frequently used by both groups was phone-based GP consultations followed by clinic-based nurse and GP visits. In terms of hospital-based services, intervention participants had, on average, fewer hospital-based physiotherapy visits, outpatient visits, inpatient nights in hospital, and accident and emergency visits resulting in an inpatient stay. Conversely, there were more day case hospital visits and hospital-based mental health services attendances for intervention versus control participants, on average.

The largest cost differences resulted from hospital-based services, medication costs and the intervention cost itself. The medication costs were estimated to be lower for intervention group participants than those in the control group (-£68.90; 95% CI -£77.19 to -£60.62). The intervention was estimated to cost £187.49 per participant, comprising the cost of training (£31.92), equipment (£12.50) and of running the course of 12 classes (£142.25), which incorporated both online and face-to-face delivery methods in the base-case analysis; a sensitivity analysis explored the different delivery modes. Online classes had higher costs associated with them than face-to-face classes, resulting in the intervention cost being £195.52 and £175.44 for online and face-to-face scenarios, respectively.

The total mean costs for the intervention group over the 12-month time horizon were higher than in the control group: £1,964.96 (95% CI £1,882.38 to £2,047.55) versus £1,885.69 (95% CI £1,795.53 to £1,975.85). Participants in the intervention group had a greater number of mean QALYs than control participants, 0.731 (95% CI 0.724 to 0.738) versus 0.708 (95% CI 0.700 to 0.716). Overall, the incremental analysis identified a cost increase of £80.85 (95% CI £76.73 to £84.97) and an additional 0.0178 QALYs (95% CI 0.0175 to 0.0180) for the intervention when compared to control ( Table 5). The resulting incremental cost-effectiveness ratio of £4,546 per QALY falls under the UK NICE willingness-to-pay threshold of £20,000 per QALY. Also using this threshold, the incremental net monetary benefit was £274.85 (95% CI £268.29 to £281.41); a positive value indicates the intervention is cost-effective when compared with usual care. Point estimates generated from the analyses were found to populate all four quadrants of the cost-effectiveness plane, indicating uncertainty in the findings (extended data ^20^). Cost-effectiveness acceptability curves illustrated a 79% probability of the intervention being cost-effective at the £20,000 per QALY threshold (extended data ^20^). The cost-effectiveness findings remained robust to the sensitivity analyses undertaken, with the incremental cost per QALY remaining below the £20,000 per QALY threshold for all analyses ( Table 5).

**Table 5.** Cost-utility analysis results.

Sensitivity Analysis (SA)	Incremental mean cost (95% CI) ^a^	Incremental mean QALYs (95% CI) ^a^	ICER (£): cost per QALY	Probability cost- effective, £20,000/QALY
Base case (MI), NHS perspective	80.85 (76.73, 84.97)	0.0178 (0.0175, 0.0180)	£4546.03	79%
SA1: complete case analysis	96.08 (-360.00, 552.16)	0.0237 (-0.0136, 0.0611)	£4049.20	77%
SA2: personal expenses	116.94 (112.72, 121.15)	0.0170 (0.0168, 0.0172)	£6883.00	74%
SA3: face-to-face yoga courses only	68.80 (64.69, 72.92)	0.0178 (0.0175, 0.0180)	£3868.60	81%
SA4: online yoga courses only	88.88 (84.77, 93.00)	0.0178 (0.0175, 0.0180)	£4997.65	79%
SA5: medication cost excluded	149.23 (145.14, 153.32)	0.0178 (0.0175, 0.0180)	£8395.54	73%
SA6: removing age & gender	28.31 (24.35, 32.27)	0.0184 (0.0182, 0.0186)	£1537.66	85%

^a^ Difference between groups (intervention – control), with a bivariate model using seemingly unrelated regression used to estimate 95% CIs. All analyses are adjusted for the following covariates: baseline utility, age, gender and study site (with the exception of SA6)

### Process evaluation

This section will offer a very brief overview of some core results that help enable reflection on the effectiveness outcomes of the trial. The process evaluation results will be reported in full elsewhere. Initial interviews were conducted with yoga participants (n=25), usual care participants (n=2), trial decliners (n=1) and yoga teachers (n=11). Follow-up interviews were conducted with yoga participants (n=15) between three and eight months post-intervention and yoga teachers (n=3) within three months post-intervention. Observations were conducted of standardisation training sessions (n=2) and yoga classes (n=10). The demographics of the process evaluation yoga participants are broadly reflective of the wider trial cohort. However, in line with qualitative research methodology, certain demographics were purposely targeted. For example, interviewees typically had a higher number of conditions, for example, 28% (n=7) had six or more compared to 4.5% in yoga arm of trial.

Participant engagement with the trial was sustained throughout the life of the trial. Initially, participants agreed to take part for a range of reasons: potential benefit to health, invitation provided an opportunity for exercise, curiosity about yoga, and altruism. Nearly all demonstrated a clear desire to make some form of change in their health and wellbeing. Over time, participants actively engaged with the classes. The GYY style of yoga – delivered both face-to-face and online – was viewed as a suitable and safe form of physical movement for people with varying health issues. However, engagement in social time, after the formal movement and meditative aspects of the class, was variable. In part, this tied to the presence, or not, of a desire to socialise with others. Relatedly, engagement with home practice was also variable, with reported adherence ranging from zero through to daily practice. Engagement was mediated by the perceived biopsychosocial benefit gained from practice.

Irrespective of their level of MLTC, most participants viewed their health as good. It was the presentation, not the presence, of a health condition that determined its impact on them. Participants and yoga teachers noted a good level of functional ability. Participants routinely reported low symptom severity and good wellbeing. The majority of participants viewed GYY as a form of gentle exercise. Only two interview participants found the class content physically challenging. Several yoga teachers also noted that the GYY style of yoga may not be challenging enough for the more functionally able individuals they worked with. Several trial participants also queried the inclusion criteria of the trial, feeling that they should have been recruited based on health status rather than age.

Some participants noted no impact of yoga on their health or lifestyle. This was primarily associated with describing a state of good health and physical activity when entering the trial, with the physical yoga content not being at a level capable of providing additional functional or sustained benefits. Some described a modest impact on aspects of physical and psychological health and self-management benefits. This included improvements in muscle strength, reduction in pain and stiffness, greater postural awareness, mobility and balance coordination alongside improved management of sleep, emotional, and mental wellbeing. Some described a transformative impact of GYY, with yoga – both GYY and more physically challenging yoga styles – becoming integral to their daily life.

## Discussion

### Principal findings

This randomised trial evaluated the effectiveness and cost-effectiveness of offering a 12-week chair-based yoga programme in addition to usual care in older adults with MLTC. The results show no statistically or clinically significant effect from offering the GYY programme in respect of HRQOL measured using the EQ-5D-5L, which was the primary outcome. Another measure of HRQOL, the PROMIS-29, showed similar findings; that is, all the PROMIS outcomes showed no evidence of effect except for pain interference and pain intensity, which showed small improvements associated with the intervention. There were no statistically significant between-group differences in the secondary outcomes of depression, anxiety, loneliness, or falls. No serious, related adverse events were reported. The economic evaluation showed that the intervention was associated with additional costs of £80.85 per participant and generated an additional 0.0178 QALYs per participant, on average, compared with usual care. The combined effect was that the GYY programme was likely to be cost-effective at the usual thresholds for willingness to pay. The process evaluation interviews highlighted that participants viewed GYY as a suitable and safe activity for older people with varying health issues. The perceived impact of the GYY programme ranged from minimal to transformative. For some participants, there was no impact on their health or lifestyle. For others, yoga became an integral part of their life and they felt it generated a broad range of benefits including improvements in physical function, joint pain and stiffness, and mental wellbeing.

### Comparison with previous studies

As this is the first adequately powered trial of yoga for older adults with MLTC, direct comparisons with other trial data are limited. However, systematic reviews on similar questions have reported mixed findings ^33– 35^. Our trial is most closely aligned to the systematic review by Tulloch and colleagues ^34^, which reported that ‘physical’ yoga interventions delivered to people aged 60 years and older increased HRQOL (standardised mean difference [SMD] 0.51, 95% CI 0.25 to 0.76) and mental wellbeing (SMD 0.38, 95% CI 0.15 to 0.62), but this review included a mixture of populations (none specifically with MLTC) and interventions (e.g., various yoga styles, programme duration ranging 8 to 24 weeks), with findings based on data from 12 trials and 752 participants. A Cochrane review by Smith and colleagues ^33^, showed little evidence that interventions for MLTC improved clinical outcomes or HRQOL, but this review did not include any yoga trials and a key conclusion was that further high-quality trials are needed. The comparisons presented here have limitations in their applicability. Nevertheless, the outcome of the current trial provides the best estimate of the effects of offering GYY to older people with MLTC, and specifically in the context of the UK healthcare system. The effects of yoga in this specific population should be further explored through meta-analysis once additional combinable studies have been performed.

### Strengths and limitations of the study

This trial has several strengths. It is the first adequately powered RCT of yoga for older adults with MLTC and was rigorously undertaken in line with recommended standards for individually randomised trials. The trial was prospectively registered and the protocol was published. External validity was enhanced by using broad eligibility criteria and recruiting from a range of primary care practices across England and Wales. Randomisation was conducted by a secure web-based system with concealed allocation. The intervention was standardised and delivered by 12 experienced teachers who all held a regulated qualification in Teaching GYY. Class attendance rates were good, as was adherence to home yoga practice during the intervention period. There was little evidence of control group contamination. The number of participants randomised provided sufficient power as per our sample size assumptions and there were high rates of participant follow-up over 12 months. We collected data on a range of outcomes, several of which feature in a core outcome set for MLTC trials ^36^. The two randomised groups were comparable on almost all the baseline characteristics. We performed sensitivity analyses, which confirmed the findings of our primary analysis, indicating the robustness of our results. We also conducted economic and process evaluations, both of which have been lacking in most previous studies of yoga or interventions for MLTC. The trial was reported in line with CONSORT and other relevant guidelines ^37^. Finally, an independent Trial Steering Committee helped ensure that participant safety issues were considered and that the trial was conducted as planned.

The trial also has some limitations. First, only 3.5% of invited patients were recruited. This rate of recruitment is typical of trials using this type of intervention and recruitment strategy ^38^, but raises the possibility of recruitment bias. Reassuringly, the trial participants appear reasonably representative of the wider population of older adults with MLTC when characteristics are compared with data from nationally representative datasets ^39– 41^, apart from a slight under-representation of males, non-White ethnic groups, and people with lower socioeconomic status. Second, there was a slight imbalance in gender at baseline, but this was adjusted for in a sensitivity analysis and did not change the interpretation. Third, the COVID-19 pandemic required us to change our processes for recruitment, follow-up, and intervention delivery part way through the trial. Regarding intervention delivery, the British Wheel of Yoga continue to offer a mixture of face-to-face and online GYY classes, so the mixture of course types included in this trial reflects current practice. Finally, the large number of statistical tests performed raises the possibility of false-positive findings due to multiple testing, and the fact that most outcomes were based on participant self-report raises potential for bias in this open-label trial. However, in pragmatic trials it is important to collect data for a broad range of outcomes of relevance to various stakeholders ^42^, and the consistent results across all key outcomes adds support to our interpretation of the findings.

### Implications for practice and policy

In this trial, the GYY courses were offered free of charge as if part of the National Health Service. If shown to provide benefit relative to usual care alone, the intervention could become a commissioned service and made available more widely. The findings lead us toward the somewhat paradoxical conclusion of ‘not clinically effective but probably cost-effective’. This conclusion is because the marginal costs were low which meant that the small QALY gain produced a cost per QALY of less than £20,000. The cost-effectiveness data alone may imply that the intervention should be adopted; however, it has been argued that only exceptionally should a single trial provide grounds for implementation ^43^. The UK’s NICE and similar decision-making bodies specify that, rather than being based on a single trial, economic evaluation should generally be based on the totality of the evidence established by systematic review and meta-analysis.

Outside of the trial setting, GYY classes are available to attend on a self-pay basis, either online, or face-to-face in many parts of the UK ^44^. Our findings indicate that the intervention is safe, acceptable, and in some cases highly valued in this population. Healthcare professionals or social prescribing link workers could therefore consider recommending self-funded GYY classes where it appears a ‘good fit’ with an individual’s needs and preferences. Routinely recommending GYY to older adults with MLTC would be unlikely to improve HRQOL at the population level, but a more targeted approach may provide various benefits to individuals. Given its gentle nature, GYY might be best targeted towards older adults who are frail, experience mobility restrictions, or have a greater disease burden. For such individuals, the classes might be sufficiently stimulating to provide benefit and/or act as a gateway to more challenging forms of yoga or other forms of physical activity. From a different perspective, GYY classes could be done to contribute towards achieving a healthy amount of physical activity ^45^.

## Conclusions

The offer of a 12-week chair-based yoga programme in addition to usual care was not associated with any statistically significant benefits in terms of HRQOL or key secondary outcomes. However, the intervention was safe, acceptable to most participants, valued by some, and probably cost-effective. When deciding treatment, healthcare professionals should consider individual needs and preferences and the cost-effectiveness of the intervention. Future research should include longer-term cost-effectiveness modelling and identifying subgroups of people who are most likely to benefit from this type of intervention. Further work is also needed to help build a consensus about the most appropriate eligibility criteria and outcomes to use in intervention trials for MLTC.

## Data Availability

Open Science Framework: Underlying data for ‘Effectiveness and cost-effectiveness of offering a chair-based yoga programme in addition to usual care in older adults with multiple long-term conditions: pragmatic, parallel group, open label, randomised controlled trial’. https://doi.org/10.17605/OSF.IO/P5SE6 ^20^. This project contains the following underlying data: Data file 1. GYY_anon_analysis_data.csv. (Anonymous participant data). Data file 2. GYY_anon_analysis_data.dta. (Anonymous participant data). Data file 3. GYY data dictionary.csv. (Variable descriptions for data). Data are available under the terms of the Creative Commons Zero "No rights reserved" data waiver (CC0 1.0 Universal). Open Science Framework: Extended data for ‘Effectiveness and cost-effectiveness of offering a chair-based yoga programme in addition to usual care in older adults with multiple long-term conditions: pragmatic, parallel group, open label, randomised controlled trial’. https://doi.org/10.17605/OSF.IO/P5SE6 ^20^. This project contains the following extended data: GYY SAP.pdf (Statistical Analysis Plan) GYY HEAP.pdf (Health Economics Analysis Plan) GYY Cost-effectiveness analysis methods.pdf (additional cost-effectiveness analysis methods and results) Data are available under the terms of the Creative Commons Zero “No rights reserved” data waiver (CC0 1.0 Public domain dedication). Open Science Framework: CONSORT and CHEERS checklists for ‘Effectiveness and cost-effectiveness of offering a chair-based yoga programme in addition to usual care in older adults with multiple long-term conditions: pragmatic, parallel group, open label, randomised controlled trial’. https://doi.org/10.17605/OSF.IO/P5SE6 ^20^. Data are available under the terms of the Creative Commons Zero “No rights reserved” data waiver (CC0 1.0 Public domain dedication).
