# Supplementary material for: Effectiveness and cost-effectiveness of offering a chair-based yoga programme in addition to usual care in older adults with multiple long-term conditions: a pragmatic, parallel group, open label, randomised controlled trial
Source: NIHR Open Res. 2024 Aug 12;3:52. Originally published 2023 Oct 11. [Version 2] doi: 10.3310/nihropenres.13465.2 (PMC11411245; doi:10.3310/nihropenres.13465.2)
Supplement: Peer review history

# Reviewer report: Reviewer response for version 2

**DOI:** 10.3310/nihropenres.14789.r32860

**Published:** 2024-10-17

**Authors:** Rod Taylor

**Affiliations:** 1 University of Glasgow, Glasgow, Scotland, UK

My compliments to authors on this very well designed, conducted and reported RCT in people with MLTCs comparing yoga plus SoC or SoC alone. Given the current importance of multimorbidity and the insufficient of current evidence-based interventions, the results are very interesting albeit not completely definitive in terms of the insufficiency of clear evidence of clinical effectiveness of this 12-week chair-based yoga intervention. The design, execution and reporting of the trial will be great interest to the research community working in MLTCs.

 The authors have provided a comprehensive response and revised the manuscript accordingly to the previous peer reviewer’s comments.

 I only have two observations/suggestions for the authors to consider:

1. Subgroups - I total agree with the authors recommendation to consider importance of identifying MLTC subgroups for future research and targeting interventions. However, it seems a missed opportunity they did not undertake/report subgroup analyses (e.g. 2 LTCs vs >2 LTCs) albeit the trial was not formally powered – such exploratory analyses of these data could assist hypothesis setting for future multimorbidity trials.
2. Possible ceiling effect – The baseline values of the PROs appear quite high/good. For example, an EQ-5D of 0.74. Therefore, the lack of statistical difference between the groups at follow up may simply be reflective of a ceiling effect in these outcomes.  Our own recent analysis from our NIHR PERFORM programme of Biobank data (paper in submission) found a mean EQ5D of <0.70 in people with ≥3 LTCs and mean baseline EQ-5D of ~0.55 in randomized feasibility trial of 60 patients with MLTCs (paper in preparation). It may be that this yoga trial may have experienced a healthy volunteer effect its recruitment. I would encourage some consideration of these related issues in the discussion.

Is the work clearly and accurately presented and does it cite the current literature?

Yes

If applicable, is the statistical analysis and its interpretation appropriate?

Yes

Are all the source data underlying the results available to ensure full reproducibility?

Yes

Is the study design appropriate and is the work technically sound?

Yes

Are the conclusions drawn adequately supported by the results?

Yes

Are sufficient details of methods and analysis provided to allow replication by others?

Yes

Reviewer Expertise:

exercise-based rehabilitation; trial design and methodology

I confirm that I have read this submission and believe that I have an appropriate level of expertise to confirm that it is of an acceptable scientific standard.

# Reviewer report: Reviewer response for version 2

**DOI:** 10.3310/nihropenres.14789.r32777

**Published:** 2024-09-24

**Authors:** Harry H.X. Wang

**Affiliations:** 1 Sun Yat-Sen University, Guangzhou, China

Tew and colleagues conducted a pragmatic, parallel-group, multi-site, open-label, randomized controlled trial to assess the effectiveness and cost-effectiveness of Gentle Years Yoga© (GYY), a chair-based yoga programme on top of usual care for adults aged 65 and older with two or more long-term conditions in England and Wales. The primary outcome of the study was health-related quality of life (HRQOL), measured using the EQ-5D-5L utility index score over a 12-month period. The results indicated that, despite little evidence of significant between-group differences in HRQOL or key secondary outcomes, the 12-week group-based GYY programme demonstrated safety, acceptability, and probable cost-effectiveness. 

 As shown in this well-written paper, the strengths of the work include the careful design of the trial with adequate statistical power, patient and public involvement, detailed methodological descriptions, and rigorous analysis of data with sufficient adjustments for confounding. 

 Minor comments –

 In the Results section, it was noted that "at baseline, three quarters of participants (n=339, 74.7%) said they would prefer to be allocated to the intervention group rather than usual care alone". Given the moderate to strong interest in yoga exercise among participants, I wonder whether there was any possibility that those in the usual care group might occasionally or unintentionally seek additional information about the knowledge and practice of yoga on their own, although I agree that the control group contamination was minimal in the present study. 

 Since one of the objectives of the chair-based GYY style of yoga was to "improve muscle strength, flexibility, balance, ...", I wonder whether the measure of muscle strength may be included as a secondary outcome to ascertain the direct impact of the yoga intervention on participants in the intervention group. Existing evidence worldwide has established a link between muscle strength and health outcomes in older adults. 

 In Figure 3, the confounding factors that have been adjusted for in the measurement of the EQ-5D-5L utility index score were not clearly indicated. The word "for" appears to be missing in the sentence "Sensitivity analysis 1 ... with further adjustment (for) ..." in the footnote to Table 3.

 Overall, I agree with the other two reviewers that this is an excellent work that has addressed critical gaps in the current knowledge.

Is the work clearly and accurately presented and does it cite the current literature?

Yes

If applicable, is the statistical analysis and its interpretation appropriate?

Yes

Are all the source data underlying the results available to ensure full reproducibility?

Yes

Is the study design appropriate and is the work technically sound?

Yes

Are the conclusions drawn adequately supported by the results?

Yes

Are sufficient details of methods and analysis provided to allow replication by others?

Yes

Reviewer Expertise:

Primary Health Care

I confirm that I have read this submission and believe that I have an appropriate level of expertise to confirm that it is of an acceptable scientific standard.

# Reviewer report: Reviewer response for version 2

**DOI:** 10.3310/nihropenres.14789.r32545

**Published:** 2024-09-18

**Authors:** Diana Karamacoska

**Affiliations:** 1 Western Sydney University, South Penrith, New South Wales, Australia

The authors have done well to address the comments raised in the initial round of peer-reviews. I have no further comments for the authors and commend them on an efficient indexing process. The article is very well written and addresses many gaps in the literature, particularly in regards to involving patients and the public in the design and execution of nonpharmacological interventions.

Is the work clearly and accurately presented and does it cite the current literature?

Yes

If applicable, is the statistical analysis and its interpretation appropriate?

I cannot comment. A qualified statistician is required.

Are all the source data underlying the results available to ensure full reproducibility?

Yes

Is the study design appropriate and is the work technically sound?

Yes

Are the conclusions drawn adequately supported by the results?

Yes

Are sufficient details of methods and analysis provided to allow replication by others?

Partly

Reviewer Expertise:

NA

I confirm that I have read this submission and believe that I have an appropriate level of expertise to confirm that it is of an acceptable scientific standard.

# Reviewer report: Reviewer response for version 1

**DOI:** 10.3310/nihropenres.14608.r30738

**Published:** 2024-01-22

**Authors:** Diana Karamacoska

**Affiliations:** 1 Western Sydney University, South Penrith, New South Wales, Australia

This study investigated the health benefits of a 12-week chair-based yoga intervention for older adults living with long-term conditions. They found no significant impact on health outcome measures, but deemed it safe, accetable and somewhat cost-effective.  From a practical perspective, if an intervention is deemed to have non-significant impacts on a person's health, the justification to pay for that said intervention is, well, challenging. The authors did well to raise this in their discussion.

 Perhaps I've misunderstood the intervention, but was there only one class delivered each week and participants were encouraged to practice at home on the other days?  Is it possible this intervention design is not appropriate for this cohort (although there was PPI involvement in the design is it possible that more than one weekly instructor-led classes is needed to impact these individuals' understanding of yoga instructions, their ability and confidence to do it on their own, and subsequently their health outcomes)? Can you comment on this in the discussion, with regards to yoga protocols involving older adults with different health issues?     

 Can the authors provide the interview schedule or guide in the supplementary materials to apprecaite what was discussed in the process evaluation?  The analysis of this qualitative data is also rather vague - can you please explain how the data were analysed?

 Overall, the paper is well written and continues to build on the evidence-base for yoga interventions.

Is the work clearly and accurately presented and does it cite the current literature?

Yes

If applicable, is the statistical analysis and its interpretation appropriate?

I cannot comment. A qualified statistician is required.

Are all the source data underlying the results available to ensure full reproducibility?

Yes

Is the study design appropriate and is the work technically sound?

Yes

Are the conclusions drawn adequately supported by the results?

Yes

Are sufficient details of methods and analysis provided to allow replication by others?

Partly

Reviewer Expertise:

I have expertise in conducting brain health research, lifestyle medicines and complementary interventions.

I confirm that I have read this submission and believe that I have an appropriate level of expertise to confirm that it is of an acceptable scientific standard, however I have significant reservations, as outlined above.

# Response

**Published:** 2024-05-11

**Authors:** Garry Tew

**Affiliations:** York St John University, York, England, UK

**This study investigated the health benefits of a 12-week chair-based yoga intervention for older adults living with long-term conditions. They found no significant impact on health outcome measures, but deemed it safe, acceptable and somewhat cost-effective.  From a practical perspective, if an intervention is deemed to have non-significant impacts on a person's health, the justification to pay for that said intervention is, well, challenging. The authors did well to raise this in their discussion.**

 Thank you for reviewing our manuscript and for your useful feedback.

**Perhaps I've misunderstood the intervention, but was there only one class delivered each week and participants were encouraged to practice at home on the other days?  Is it possible this intervention design is not appropriate for this cohort (although there was PPI involvement in the design is it possible that more than one weekly instructor-led classes is needed to impact these individuals' understanding of yoga instructions, their ability and confidence to do it on their own, and subsequently their health outcomes)? Can you comment on this in the discussion, with regards to yoga protocols involving older adults with different health issues?**

 You haven’t misunderstood; intervention group participants were offered 12 weekly classes and encouragement to practice yoga independently on most days. One interpretation of our findings could indeed be that the ‘dose’ of yoga was insufficient to have a perceivable or measurable impact. In the absence of definitive guidance on programme duration, frequency or intensity, we were guided by previous studies and routine practice, as well as an intention to test a pragmatic and scalable version of the intervention. Twelve weeks is a commonly used intervention duration in yoga trials (E.g., Elwy et al., 2014) and the mixture of weekly group classes with encouragement to perform regular home practice reflected how GYY is typically delivered outside of the trial. We have decided not to amend the manuscript because the HTA will include an expanded discussion on the interpretation of findings.

 Elwy AR, Groessl EJ, Eisen SV, Riley KE, Maiya M, Lee JP, et al. A systematic scoping review of yoga intervention components and study quality. Am J Prev Med 2014;47(2):220–32.

**Can the authors provide the interview schedule or guide in the supplementary materials to appreciate what was discussed in the process evaluation?  The analysis of this qualitative data is also rather vague - can you please explain how the data were analysed?**

 The interview data were analysed according to standard procedures of qualitative analysis. Further details of the qualitative component (methods and results) will be further reported in the HTA report and separate manuscripts.

**Overall, the paper is well written and continues to build on the evidence-base for yoga interventions.**

 Thank you.

# Reviewer report: Reviewer response for version 1

**DOI:** 10.3310/nihropenres.14608.r30667

**Published:** 2023-11-17

**Authors:** Julia Loewenthal

**Affiliations:** 1 Medicine, Brigham and Women's Hospital, Boston, Massachusetts, USA

This is an ambitious, rigorous, and well-reported randomized controlled trial that fills a gap in the literature. There are no previously published high-quality RCTs of this scale of a yoga-based intervention in older adults with multiple chronic conditions. The authors not only report on clinical effectiveness, but also performed a cost-effectiveness analysis. In addition, they report qualitative data from participants. Overall, this is a very strong study that meets a need in this area of research.

 Abstract: the manuscript mentions results of qualitative data. I would at least mention this in the Results section to guide the reader.

 The plain English summary is clear.

 The Introduction is well-written and cites relevant research in this area, including research from exercise literature (beyond just yoga). The Methods includes review by appropriate ethical board and Methods and Results are reported according to the CONSORT and CHEERS guidelines.

 For participant eligibility (page 5-6 and Table 2), how were long-term conditions selected?

 One drawback of the intervention delivery is that delivery was changed from face-to-face to a mixture of face-to-face and online due to the COVID-19 pandemic. This was understandably unexpected. This could have impacted loneliness and some psychological health outcomes, but delivery was equivalent in intervention and control groups and this change was acknowledged in the Discussion. In addition, it does reflect real-world practice, as mentioned by the authors. One minor issue is that it would be helpful to know if participants were given equipment or provided with any technical support for online practice.

 Figure 2-- interestingly, more participants withdrew from the trial early in the intervention arm and later in the control arm. Were interviews conducted with participants who withdrew? It might be helpful to understand if this was due to allocation preference (pg. 9), musculoskeletal pain as mentioned on pg. 13, or other reasons.

 The resource use point on pg. 14 is under-emphasized, particularly the fact that intervention participants had lower rates of hospitalization and emergency service use. This would be helpful to briefly report in the Abstract and include in Discussion. 

 The cost-effectiveness analysis strengthens this trial and fills a significant gap in the literature.

 Hopefully the qualitative data will be reported separately as mentioned on pg. 17 and analyzed rigorously using qualitative research methods.

 A major point mentioned by the authors in the Discussion (pg. 17-18) is that GYY may be effective for certain older adults. Notably, there is no data reported on baseline functional or frailty status of the participants. GYY seems to be a fairly gentle chair-based yoga sequence that may not provide additional QOL or physical health benefits to relatively physically able and/or robust older adults. This may be a consideration for future work since older adults can have multiple chronic conditions but have robust physical function status and no disability (defined by ADLs/iADLs).

Is the work clearly and accurately presented and does it cite the current literature?

Yes

If applicable, is the statistical analysis and its interpretation appropriate?

I cannot comment. A qualified statistician is required.

Are all the source data underlying the results available to ensure full reproducibility?

Yes

Is the study design appropriate and is the work technically sound?

Yes

Are the conclusions drawn adequately supported by the results?

Yes

Are sufficient details of methods and analysis provided to allow replication by others?

Yes

Reviewer Expertise:

Geriatrics, aging, yoga, mind-body medicine, education

I confirm that I have read this submission and believe that I have an appropriate level of expertise to confirm that it is of an acceptable scientific standard.

# Response

**Published:** 2024-05-11

**Authors:** Garry Tew

**Affiliations:** York St John University, York, England, UK

**This is an ambitious, rigorous, and well-reported randomized controlled trial that fills a gap in the literature. There are no previously published high-quality RCTs of this scale of a yoga-based intervention in older adults with multiple chronic conditions. The authors not only report on clinical effectiveness, but also performed a cost-effectiveness analysis. In addition, they report qualitative data from participants. Overall, this is a very strong study that meets a need in this area of research.**

 Thank you for reviewing our manuscript and for your useful feedback.

**Abstract: the manuscript mentions results of qualitative data. I would at least mention this in the Results section to guide the reader.**

 We have added the following sentence at the end of the results section in the abstract: “The intervention was acceptable to most participants and perceived as useful by some.”

**The plain English summary is clear.**

 Thank you.

**The Introduction is well-written and cites relevant research in this area, including research from exercise literature (beyond just yoga). The Methods includes review by appropriate ethical board and Methods and Results are reported according to the CONSORT and CHEERS guidelines.**

 Thank you.

**For participant eligibility (page 5-6 and Table 2), how were long-term conditions selected?**

 The list of eligible long-term conditions was based mostly on the conditions included in the NHS Quality and Outcomes Framework (QOF version 31.0) with some other conditions added following discussions amongst the trial management group.

**One drawback of the intervention delivery is that delivery was changed from face-to-face to a mixture of face-to-face and online due to the COVID-19 pandemic. This was understandably unexpected. This could have impacted loneliness and some psychological health outcomes, but delivery was equivalent in intervention and control groups and this change was acknowledged in the Discussion. In addition, it does reflect real-world practice, as mentioned by the authors. One minor issue is that it would be helpful to know if participants were given equipment or provided with any technical support for online practice.**

 Prior to online courses commencing, participants were sent several props (e.g., resistance bands, tennis ball, block) in an individual equipment pack to use in their classes. Preliminary one-to-one Zoom meetings were also held between to yoga teachers and their participants to discuss equipment set-up, health issues, and any course queries.

**Figure 2-- interestingly, more participants withdrew from the trial early in the intervention arm and later in the control arm. Were interviews conducted with participants who withdrew? It might be helpful to understand if this was due to allocation preference (pg. 9), musculoskeletal pain as mentioned on pg. 13, or other reasons.**

 Interviews were not conducted with participants who withdrew.

**The resource use point on pg. 14 is under-emphasized, particularly the fact that intervention participants had lower rates of hospitalization and emergency service use. This would be helpful to briefly report in the Abstract and include in Discussion.**

 Thank you. We have decided not to amend the manuscript in response to this comment because the abstract is already up to the maximum word limit and an expanded discussion of the economic evaluation will be included in the full HTA report.

**The cost-effectiveness analysis strengthens this trial and fills a significant gap in the literature.**

 Thank you, we agree.

**Hopefully the qualitative data will be reported separately as mentioned on pg. 17 and analyzed rigorously using qualitative research methods.**

 The qualitative data will be further reported in the HTA report and separate manuscripts. The interview data were analysed according to standard procedures of qualitative analysis.

**A major point mentioned by the authors in the Discussion (pg. 17-18) is that GYY may be effective for certain older adults. Notably, there is no data reported on baseline functional or frailty status of the participants. GYY seems to be a fairly gentle chair-based yoga sequence that may not provide additional QOL or physical health benefits to relatively physically able and/or robust older adults. This may be a consideration for future work since older adults can have multiple chronic conditions but have robust physical function status and no disability (defined by ADLs/iADLs).**

 Thank you for this comment.
